# Psychosocial determinants of healthcare personnel’s willingness to carry real-time locating system tags during daily inpatient care in hospital managing COVID-19 patients: insights from a mixed-methods analysis

**DOI:** 10.1093/jamiaopen/ooaa072

**Published:** 2021-02-05

**Authors:** Huiling Guo, Zhilian Huang, Jeanette Y P Yeo, Yinchu Wang, Angela Chow

**Affiliations:** 1 Department of Clinical Epidemiology, Office of Clinical Epidemiology, Analytics, and Knowledge, Tan Tock Seng Hospital, Singapore; 2 Lee Kong Chian School of Medicine, Nanyang Technological University, Singapore

**Keywords:** real-time locating system, contact tracing, hand hygiene, healthcare personnel, technology acceptance

## Abstract

**Objective:**

Real-time locating systems (RTLS) enable contact tracing and hand hygiene reminders, to improve hospital safety. Successful implementation requires healthcare personnel (HCP) to carry RTLS tags continuously. We assessed for determinants of HCP’s willingness to use RTLS tags during routine inpatient care, and evaluated concerns using mixed-methods analysis.

**Materials and Methods:**

We conducted a cross-sectional study in the 330-bed purpose-built National Centre for Infectious Diseases in Singapore, from January 15 through February 4, 2020. The anonymous survey comprised 24 questions based on constructs from behavioral models and an open-ended question. Principal component analysis was performed to derive the latent factor structure applied in the multivariable logistic regression analysis. Concerns were analyzed using thematic analysis.

**Results:**

Of 260 HCP (nurses [40.8%], ancillary and administrative staff [23.1%], allied health professionals [18.5%], and physicians [17.7%]), 75% were willing to use the RTLS tag. After adjusting for age, gender, healthcare professional group, and duration of practice, the acceptance of the use of the RTLS tag (adjusted OR 11.28 [95% CI 4.39–29.00], *P* < .001) was highly associated with the willingness to use the RTLS tag. HCP who perceived the tag to be easy to use (adjusted OR 2.80 [95% CI 1.37–5.72], *P* = .005), were also more willing to use the tag. HCP were willing to carry the RTLS tag for the purpose of contact tracing despite privacy concerns.

**Conclusion:**

More communications on the intentions and data protection standards of the RTLS, and accessory enhancements for HCP’s convenient and sustained use of the RTLS tag are crucial, to optimize RTLS’s usefulness during the COVID-19 pandemic.

## BACKGROUND AND SIGNIFICANCE

Real-time locating systems (RTLS) have a wide range of applications in healthcare, and a global market forecasted to quadruple to US$6.4 billion by 2027.^1^ The market growth in RTLS represents its potential in enhancing healthcare delivery and improving patient safety. An RTLS is an indoor positioning application that can locate a person or object tagged with radiofrequency identification (RFID) in real-time.^2^ Some of its capabilities include tracking hospital assets,^3–5^ monitoring patient safety for falls prevention,^6^^,^^7^ monitoring hand hygiene compliance for infection prevention and control,^8^^,^^9^ and contact tracing during an outbreak.^10^ Studies on staff-worn RFID tags have shown promises in the accuracy and efficiency of RTLS technology for contact tracing compared with conventional methods.^10–12^ The ability to identify potentially exposed healthcare personnel (HCP) to an infectious patient is crucial for preventing nosocomial transmission.^13^ The ongoing COVID-19 pandemic has highlighted the increased risk of HCP to nosocomial infections.^14^ Contact tracing is the key strategy for preventing the further transmission of COVID-19 and the adoption of emerging technologies can greatly enhance the efficiency of contact tracing.^15^ However, the potential of RTLS for contact tracing can only be realized with its successful implementation from HCP’s willingness to adopt and use it.^12^^,^^16^

The success of implementing a novel technology in an organization hinges on many factors, such as cost-efficiency, legal requirements, organizational culture, ease of adoption, and user acceptance of the new technology.^16–19^ User acceptance is the linchpin of technology adoption and implementation success,^20^ but ensuring compliance in technology adoption can be a challenge.^16^ HCPs have to weigh the organization’s needs against their rights to privacy.^16^^,^^21^^,^^22^ Failure to address concerns over job insecurity, undesired scrutiny, and privacy loss,^21^ can lead to staff resentment, underutilization, and even sabotage of the new technology.^20^ A handful of studies assessing HCP’s attitudes and perceptions on the use of RFID tags have been conducted in emergency departments,^16^^,^^18^^,^^21^ with no study to date has been carried out in inpatient settings. Furthermore, studies on the acceptance of RTLS in healthcare have focused largely on the views of the hospital management^17^^,^^23^^,^^24^ but have rarely assessed for the acceptance of HCP who are the actual users of the technology,^25^ much less evaluate the differences in the perceptions of different categories of HCP. Understanding and addressing the concerns of specific HCP groups is crucial for the successful implementation and sustained use of RTLS technologies in inpatient areas managing infectious patients.

We, therefore, sought to assess the psychosocial determinants of HCP’s willingness to use RTLS tags routinely during inpatient care for infectious disease patients and to compare and contrast the influencing factors in different HCP groups (physicians, nurses, allied health professionals, and ancillary and administrative staff), as well as appreciate the experience and concerns of HCP on the use of RTLS tags, using a mixed-methods study design.

## MATERIALS AND METHODS

### Study setting and population

**Figure 1.**
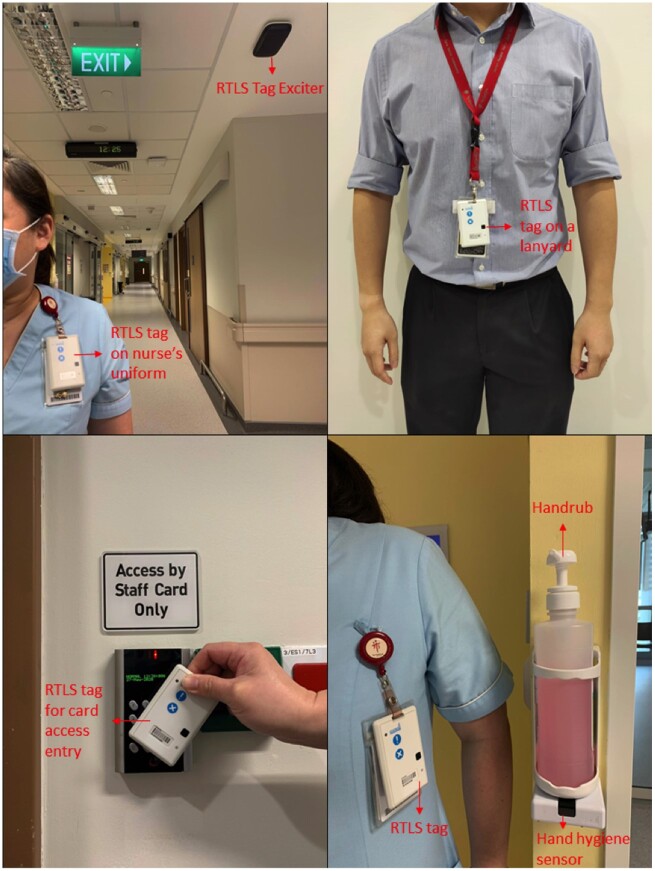
Photographs of the RTLS tag, the manner it is carried by staff, and its functions.

The National Centre for Infectious Diseases (NCID) in Singapore which is co-located with the 1600-bed multidisciplinary Tan Tock Seng Hospital, is a 330-bed purpose-built facility for the clinical management of highly infectious emerging infectious diseases including COVID-19, MERS, and Ebola. Since its official opening in September 2019, the RTLS was incorporated into NCID’s work processes.^26^ Healthcare staff working in the NCID are issued personalized RTLS tags that serve as entry and exit access cards to the premises, for location tracking for the purposes of contact tracing during an outbreak, as well as to provide visual and auditory nudges to enhance hand hygiene compliance (Figure 1). The RTLS tags are 8 cm (length) by 5 cm (width) by 0.8 cm (thickness), and weigh 38 g. The waterproof low-powered tag has a rechargeable battery life of 2 months and is enabled with tag-to-tag active RFID technology that leverages both RFID and Wi-Fi for triangulation of location (©CADI Scientific). The study was initiated just as the first case of COVID-19 was confirmed at NCID on January 23, 2020. During the study period, NCID was anticipating a surge in admissions due to COVID-19 infections. All physicians, nurses, allied health professionals (AHPs), and ancillary and administrative staff (AAS) who were issued with an RTLS tag and working in the NCID inpatient wards during the 3-week study period, January 15–February 4, 2020, were invited to participate in the study. AAS included healthcare assistants who provided support for nursing activities in patient care, patient service associates who provided administrative support for the inpatient wards, and housekeeping personnel.

### Survey administration

This is a concurrent embedded mixed-methods study, with qualitative insights being explored to complement the findings from the quantitative survey.^27^ An anonymous self-administered questionnaire was distributed to eligible staff via the respective administrative staff of clinical departments and wards with the approval of the heads of department and chiefs of services. Completed survey forms were deposited into sealed collection boxes placed at convenient locations in staff offices.

The survey instrument comprised 24 questions (on a 5-point Likert-scale) on the perceptions and attitudes towards the use of RTLS tags, based on constructs from Davis’ Technology Acceptance Model,^28^ Azjen’s Theory of Planned Behavior,^29^ and Venkatesh’s Unified Theory of Acceptance and Use of Technology (UTAUT) model.^30^ Davis’s Technology Acceptance Model (TAM) posits that the actual use of a new technology can be explained by the user’s behavioral intention to use the technology.^28^ This model was adapted from Azjen’s Theory of Planned Behaviour (TPB), which assumes that the behavioral intention to complete a task is influenced by the attitude (perceived outcome), subjective norm (others’ perception of the individual), and perceived behavioral control (ease of completing the task) of the task.^29^ Many scholarly works have extended the TAM to explain usage behavior and to include other factors that could influence users’ attitudes towards the acceptance of a new technology, including technology anxiety, privacy risk harm, cultural and social influences, and the availability of support and resources.^31–33^ Therefore, we developed questions in the survey instrument based on the constructs of performance expectancy (perceived usefulness of RTLS), effort expectancy (perceived ease of use of RTLS), social influence (perceived social norm), and perceived privacy risk harm (concerns about personal privacy).

Additionally, the questionnaire incorporated two questions on the current manner that staff carried their RTLS tags and the common challenges faced with the daily use of the RTLS tag, based on earlier observations from an ethnographic study, and an open-ended question to derive qualitative insights to enhance the quantitative results obtained on staff’s experience and concerns with the RTLS tag. The survey questionnaire also collected information on the demographics and designation of the staff, their knowledge of the functions of the RTLS tag, and their frequency of use of the tag. The questionnaire was piloted with ten physicians, nurses, AHPs, and AAS who had RTLS tags but did not work in the NCID inpatient wards for clarity of language, understanding of questions, and flow of the questions. Based on their feedback, minor edits were made to improve the understanding of the questions.

### Data analysis

Means [standard deviations (SD)] were computed for each of the 24 questions on a 5-point likert scale and compared between healthcare professional groups. One-way ANOVA with Tukey’s honestly significant difference test was used to compare the differences between group means. Willingness to use the RTLS tag whilst working in NCID was defined as having a response of “Agree” or “Strongly Agree” to the question.

#### Principal component analysis

Using the 24 likert-scale questions, we performed principal component analysis with promax rotation to derive the latent factor structure that was later applied in the multivariable logistic regression analysis to assess for independent factors associated with willingness to use the RTLS tag in NCID. Factor loadings of less than 0.35 were removed from the analysis. Internal consistencies were assessed using Cronbach's alpha. A score of more than 0.7 is considered as good. Latent factors derived from the principal component analysis were subsequently fitted into the multivariable regression model.

#### Multivariable regression analysis

Stepwise regression was used to select for variables in the final multivariable logistic regression model. To adjust for potential confounding, socio-demographic factors determined *a priori* from the literature review to be associated with the willingness to use RFID technology (such as gender and age), and healthcare professional group and duration of practice were also included in the model. Statistical analyses were conducted in Stata version 15.0 (StataCorp LLC, College Station, TX, USA).

#### Qualitative analysis

Theoretical thematic analysis was conducted on the open-ended responses to explore factors associated with HCP experience with using the RTLS tag and their concerns about it. Two coders (J.Y.P.Y. and Y.W.) independently coded the responses deductively using constructs from the UTAUT model^30^ and any discrepancies in codes were subsequently reviewed for consensus through discussions with a third study team member. Major semantic themes were identified and any new emerging themes not classified by the model were also included in the analysis. Themes and sub-themes were subsequently quantified in the analysis, with representative quotes presented.

## RESULTS

### Characteristics of the study population

A total of 260 out of 361 (72%) eligible HCP completed the survey. Nurses (40.8%) and AAS (23.1%) formed the majority, followed by AHPs (18.5%) and physicians (17.7%) (Table 1). There was a preponderance of females among nurses (96%) and AHPs (83.7%). Physicians tended to be older and more clinically experienced, with 40% aged >40 years and almost 60% having practiced for >10 years. Most respondents, in particular AAS (96.6%), had worked in inpatient wards in NCID for more than 20 days in a month. Physicians were least aware of the technologies employed for real-time location tracking and behavior monitoring (91.1%), and also least likely to carry their RTLS tag all the time while working in the NCID (78.3%). The majority (75%) of HCP have expressed willingness to use the RTLS tag when working in the NCID, with AAS being the most willing (90%). When carrying the RTLS tag, AAS most preferred wearing it on the lanyard (75.0%, *P* < .001), whilst nurses most preferred attaching it with a clip to their uniforms (84.0%, *P* < .001) and female physicians placing it in the bag (23.9%, *P* < .001) (data not shown).

**Table 1.** Characteristics of Study Participants

	Total (*N* = 260)	Physicians (*N* = 46)	Nurses (*N* = 106)	Allied health professionals (*N* = 48)	Ancillary and administrative staff (*N* = 60)
Gender, *N* (%)					
Female	189 (77.1)	22 (51.2)	96 (96.0)	36 (83.7)	35 (59.3)
Age, in years, *N* (%)					
21–30	113 (43.6)	10 (21.7)	50 (47.6)	25 (52.1)	28 (46.7)
31–40	85 (32.8)	18 (39.1)	38 (36.2)	17 (35.4)	12 (20.0)
41–50	38 (14.7)	10 (21.7)	11 (10.5)	5 (10.4)	12 (20.0)
>50	23 (8.9)	8 (17.4)	6 (5.7)	1 (2.1)	8 (13.3)
Duration of practice as healthcare professional, *N* (%)					
>10 years	83 (33.6)	26 (57.8)	39 (38.2)	14 (33.3)	4 (6.9)
Duration worked in NCID building in a month, in days, *N* (%)					
0–10	46 (18.3)	22 (47.8)	6 (5.8)	17 (38.6)	1 (1.7)
11–20	45 (17.9)	2 (4.4)	36 (35.0)	6 (13.6)	1 (1.7)
>20	160 (63.8)	22 (47.8)	61 (59.2)	21 (47.7)	56 (96.6)
Awareness of technologies employed for real-time location tracking and behavior monitoring, *N* (%)					
Aware	243 (96.1)	41 (91.1)	100 (96.2)	44 (95.7)	58 (100)
Frequency of carrying RTLS tag when working in NCID building, *N* (%)					
All the time	242 (93.4)	36 (78.3)	102 (97.1)	45 (93.8)	59 (98.3)
Willingness to use RTLS in NCID, *N* (%)					
Agree	195 (75.0)	29 (63.0)	79 (74.5)	33 (68.8)	54 (90.0)

### Awareness of RTLS tag functions

**Figure 2.**
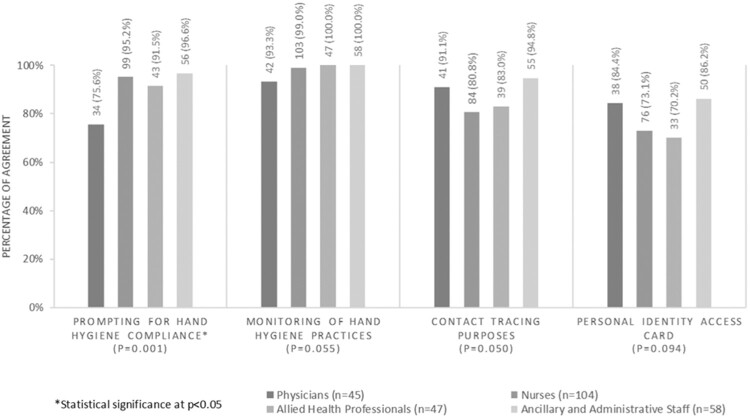
HCP’s awareness of RTLS tag functions.

HCP were generally aware of the functions of the RTLS tag although there were no statistically significant differences between HCP groups. AHPs were least aware that their RTLS tags were tagged to their individual identities (70.2%), nurses were least aware that the tags could serve as contact tracing tools (80.8%), while physicians were least aware that the RTLS tag could monitor hand hygiene compliance (93.3%) (Figure 2). However, physicians were significantly less aware that their RTLS tags could prompt for hand hygiene than other HCP (75.6%, *P* = .001).

### Factors associated with willingness to use the RTLS tag

**Figure 3.**
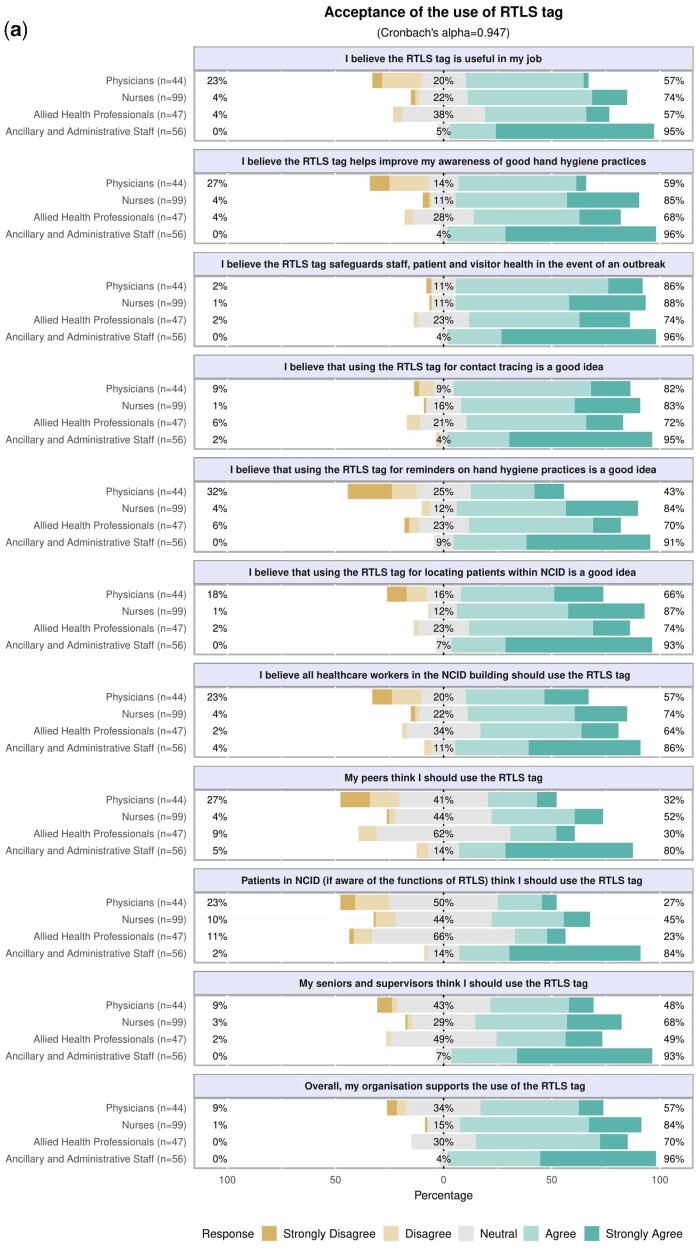
(a) Acceptance of the use of the RTLS tags. (b) Perceived ease of use of RTLS tags. (c) Privacy concerns on the use of RTLS tags.

Three psychosocial factors were identified on principal component analysis: acceptance of the use of the RTLS tag (Cronbach’s ***α* = **0.9473), perceived ease of use of the RTLS tag (***α* = **0.8841), and privacy concerns about the RTLS tag (***α* = **0.8575) (Figure 3 and Appendix 1). Physicians were less accepting of the use of RTLS tag (mean factor score −0.69 ± 1.11 SD), than nurses (0.02 ± 0.85 SD, *P* < .001) and AAS (0.79 ± 0.76 SD, *P* < .001). AAS were more likely to use the RTLS tag if they perceived that their peers, seniors, and supervisors, as well as the patients, would prefer them to use the tag (Appendix 1). Additionally, physicians were less likely to perceive that the RTLS tag was easy to use (−0.66 ± 1.15 SD) than nurses (−0.01 ± 0.88 SD, *P* < .001) and AAS (0.76 ± 0.72 SD, *P* < .001). In contrast, AAS had a significantly higher level of privacy concerns about the use of the RTLS tag (0.79 ± 0.86 SD) than physicians (−0.01 ± 1.11 SD, *P* < .001), nurses (−0.22 ± 0.88 SD, *P* < .001), and AHPs (−0.47 ± 0.75 SD, *P* < .001). After adjusting for age, gender, healthcare professional group, and duration of practice, the acceptance of the use of the RTLS tag (adjusted OR 11.28 [95% CI 4.39–29.00], *P* < .001) and perceived ease of use of the RTLS tag (adjusted OR 2.80 [95% CI 1.37–5.72], *P* = .005) were positively associated with the willingness to use the tag (Table 2). Privacy concerns with the use of the RTLS tag were not associated with HCP’s willingness to use it.

**Table 2.** Univariate and multivariable logistic regression analyses of factors associated with HCP’s willingness to use the RTLS tags in NCID

	Willing to use RTLS tag (*N *= 195)	Not willing to use RTLS tag (*N *= 65)	Unadjusted OR (95% CI)	***P***-value	Adjusted OR (95% CI)	***P***-value
Factor 1: Acceptance of the use of RTLS tag	–	–	14.71 (6.88–31.45)	<.001	11.28 (4.39–29.00)	<.001
Factor 2: Perceived ease of use of RTLS tag	–	–	4.62 (2.97–7.20)	<.001	2.80 (1.37–5.72)	.005
Factor 3: Privacy concerns on the use of RTLS tag	–	–	1.01 (0.75–1.36)	.941	0.69 (0.32–1.51)	.355
Gender, *N* (%)						
Male	46 (24.9)	10 (16.7)	Ref	–	Ref	–
Female	139 (75.1)	50 (83.3)	0.60 (0.28–1.29)	.192	0.37 (0.08–1.77)	.211
Age in years, *N* (%)						
21–30	83 (42.6)	30 (46.9)	Ref	–	Ref	–
31–40	66 (33.9)	19 (29.7)	1.26 (0.65–2.43)	.499	2.69 (0.69–10.53)	.156
41–50	27 (13.9)	11 (17.2)	0.89 (0.39–2.01)	.774	0.81 (0.13–4.99)	.819
>50	19 (9.7)	4 (6.3)	1.72 (0.54–5.46)	.360	12.43 (1.00–155.29)	.050
Designation, *N* (%)						
Physicians	29 (14.9)	17 (26.2)	Ref	–	Ref	–
Nurses	79 (40.5)	27 (41.5)	1.72 (0.82–3.60)	.154	0.49 (0.11–2.19)	.350
Allied health professionals	33 (16.9)	15 (23.1)	1.29 (0.55–3.03)	.560	1.54 (0.30–8.01)	.606
Ancillary and administrative staff	54 (27.7)	6 (9.2)	5.28 (1.88–14.84)	.002	0.73 (0.09–5.62)	.760
Duration of practice as healthcare professional, *N* (%)						
≤10 years	127 (67.9)	37 (61.7)	Ref	–	Ref	–
>10 years	60 (32.1)	23 (38.3)	0.76 (0.42–1.39)	.373	0.60 (0.14–2.56)	.486

### Experience and concerns with the use of RTLS

#### Quantitative findings

**Figure 4.**
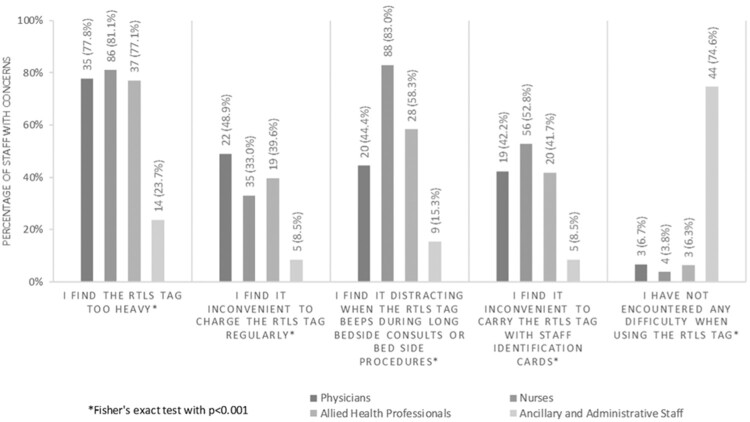
HCP’s concerns with the use of RTLS tags.

A higher proportion of nurses (81.1%) found the RTLS tag to be heavy, compared with physicians (77.8%), AHPs (77.1%), and AAS (23.7%) (*P* < .001) (Figure 4). More nurses also tended to find the RTLS tag distracting when the RTLS tag beeped during long hours of bedside care (83.0%, *P* < .001) and inconvenient to carry with the staff ID card (52.8%, *P* < .001) than other HCP groups. On the other hand, almost half of the physicians felt that it was inconvenient to charge the RTLS tag regularly (48.9%, *P* < .001), while the majority of AAS did not encounter any difficulty when using the RTLS tag (74.6%, *P* < .001).

#### Qualitative findings

Three major themes emerged from the qualitative analysis of the open-ended question on the HCP’s experience and concerns with the use of the RTLS tags: physical inconvenience, personal acceptance, and technical support (Table 3). These can be framed into the three key constructs of effort expectancy, performance expectancy, and facilitating conditions in the UTAUT model.^30^

**Table 3.** Themes, sub-themes, and representative quotes from healthcare personnel on their experience and concerns with the use of RTLS tags

				Number of feedback				
Main theme	Sub-theme	Description	Representative quotes, HCP category, years in practice	Total	Physicians(*N *= 29)	Nurses(*N *= 79)	AHPs(*N *= 33)	AAS(*N *= 54)
**(A) Physical inconvenience**	(i) Dimension	RTLS tag is heavy and bulky	“The tag is too heavy.” (AAS 0230, 5.8 years) “Tag too heavy, neck strain and not ergonomic when looking at laptop.” (AHP 0029, 5 years) “Tag is heavy, sometimes can be seen dangling down. And we have to pull it [back to] position.” (Nurse 0101, 20 years) “Card is too bulky at the moment.” (Physician 0298, 7.8 years)	27	4	18	2	3
		RTLS tag can be thinner and lighter	“Slimmer & lighter RTLS tag that can be slotted into card case.” (AAS 0213, 5.5 years) “Make it smaller, lighter, less bulky.” (AHP 0038, 1.7 years) “I hope the RTLS tag can be lighter and thinner like a normal card that is not too heavy” (Nurse 0159, 12 years) “Make it less bulky - like a card.” (Physician 0291, 20 years)	40	5	16	8	11
		RTLS tag is cumbersome to carry around	“Difficult to bring [to] go anywhere.” (AAS 0259, 2.3 years) “To combine our name card with RTLS if possible, so don't need to carry many cards together.” (Nurse 0098, 8 years) “Extra device to carry.” (Physician 0001, 28 years) “Too cumbersome to bring around” (Physician 0067, 9 years)	8	2	1	1	4
	(ii) Durability	RTLS tag easily damaged	“Easy to crack and damage.” (AAS 0273, 1.2 years) “Easily broken after dropping.” (AHP 0016, 2.5 years) “Tag often breaks apart when the screws holding it together are loose. Usually the plastic around the screw housing starts to crack.” (Physician 0008, 3 years)	37	2	0	1	34
		RTLS tag can have a protective cover	“If have any cover to protect RTLS better, can prevent [it] from any damage” (AAS 0252, 10.2 years)	6	2	0	0	4
	(iii) Length of battery life	Need for charging of RTLS tag	“Charging 2-monthly [usually forget to charge].” (Nurse 0105, 4 years) “Make it fast charging.” (Nurse 0172 , 15.4 years) “Charging [is] inconvenient.” (Physician 0299, 19.4 years) “Less need to recharge” (Physician 0310, 18 years) “It would be better if a charging port is made available in each ward so that it can be charged [up] easily” (Nurse 0140, 3.7 years)	7	3	4	0	0
	(iv) Consistency of use	Non-adherence to carrying the RTLS tag all the time	“I think main barrier for me is having to carry it or wear it all the time in clinical areas.” (Physician 0065, 15.5 years) “Many a time, they left it around on table, doesn’t track movement properly. When left RTLS in bag, and HCP see patient, may not track properly.” (AHP 0034, 5 years)	3	2	0	1	0
(B) Personal acceptance	(i) Perceived usefulness of RTLS tag	Over-sensitivity of hand hygiene sensor not useful for hand hygiene reminder	“Even though you [perform] your hand hygiene, it still beeps and it is so distracting when doing my bedside care, especially when the patient asked what was that noise.” (AAS 0137, 6.5 years) “Will still beep even when I practise[d] hand hygiene prior to speaking to [a] patient.” (AHP 0027, 2 years) “[The] RTLS start[ed] to beep when [I walked] past the RTLS sensor, even when we had not contacted a patient. Even after we have done the first moment of hand hygiene before a procedure, and we pass by the sensor […] it starts to beep until the end of the procedure, which disturbs our work.” (Nurse 0229, 3.7 years) “Can also be rather inaccurate in detecting lack of compliance to hand hygiene -> will beep even if I have performed hand hygiene.” (Physician 0298, 7.8 years)	27	7	15	2	3
		Inaudible auditory cue from tag not useful for hand hygiene reminder	“Beep sound needs to [be] louder” (AAS 0260, 2.6 years) “Maybe instead of beeping. It can vibrate.” (AHP 0023, 0.7 years) “Cannot hear beeping.” (Physician 0002, 24 years)	11	1	0	1	9
		Insensitivity of RTLS tag not useful as door access card	“Not ‘user-friendly’. When tapping RTLS, only the front side [of the tag] can [be detected] […] RTLS cannot be detected if there are staff pass[es] attach[ed] to it. Must be RTLS alone.” (AAS 0213, 5.5 years) “The other inconvenience about the RTLS tag is that only one side can be scanned, hence, I always have to make sure the tag is pointing in the correct direction.” (AHP 0030, 9 years) “To improve the sensitivity of the RTLS while accessing the lock. Accessible area is too small/had to be detect by the door lock.” (Nurse 0200, 3.6 years) “Not sensitive - does not scan properly when scanner has a plastic cover.” (Physician 0294, 5 years)	20	3	7	7	3
		Appreciate RTLS tag’s usefulness for contact tracing	“Use it only for contact tracing.” (Physician 0291, 20 years) “Agree with contact trace purpose but remove the incessant beeping please. “ (Physician 0308, 6.5 years)	6	3	0	3	0
		RTLS is too expensive a system relative to its usefulness	“Huge waste of money for hand hygiene audit purposes. HCP are better trained than requiring a beeping device that cannot be intelligent enough to really accurately detect the compliance to hand hygiene.” (Physician 0308, 6.5 years)	1	1	0	0	0
	(ii) Privacy concerns	Uncomfortable to be tracked; Invasion of privacy	“Tracing staff location, feel uncomfortable.” (Nurse 0156, 1.7 years) “Invasion of privacy.” (Physician 0001, 28 years)	2	1	1	0	0
(C) Technical Support	–	To provide better technical support	“Please provide us [with a] hotline [if] we need help” (Nurse 0091, 10 years)	3	2	1	0	0

**Appendix 1.** Comparison of psychosocial factors associated with healthcare personnel’s perceptions on the use of the RTLS tag between healthcare professional groups

	Physicians(*N *= 44)	Nurses(*N *= 99)	Allied health professionals (AHPs)(*N *= 47)	Ancillary and administrative staff (AAS)(*N *= 56)	Physicians–nurses difference *P*-value	Physicians–AHPs difference *P*-value	Physicians–AAS difference *P*-value	Nurses–AHPs difference *P*-value	Nurses–AAS difference *P*-value	AHPs–AAS difference *P*-value
Factor 1: Acceptance of the use of RTLS tag (*α* = 0.9473)	−0.69 ± 1.11	0.02 ± 0.85	−0.33 ± 0.79	0.79 ± 0.76	<.001	.223	<.001	.108	<.001	<.001
Q1. I believe the RTLS tag is useful in my job	3.33 ± 0.94	3.84 ± 0.81	3.63 ± 0.73	4.67 ± 0.60	.001	.249	<.001	.398	<.001	<.001
Q2. I believe the RTLS tag helps improve my awareness of good hand hygiene practices	3.30 ± 1.09	4.12 ± 0.86	3.81 ± 0.79	4.68 ± 0.54	<.001	.018	<.001	.144	<.001	<.001
Q3: I believe the RTLS tag safeguards staff, patient and visitor health in the event of an outbreak	3.98 ± 0.68	4.22 ± 0.72	3.94 ± 0.76	4.67 ± 0.54	.189	.992	<.001	.085	<.001	<.001
Q6: I believe that using the RTLS tag for contact tracing is a good idea	3.89 ± 0.85	4.13 ± 0.73	3.79 ± 0.82	4.60 ± 0.64	.299	.919	<.001	.057	.001	<.001
Q7: I believe that using the RTLS tag for reminders on hand hygiene practices is a good idea	3.09 ± 1.33	4.11 ± 0.79	3.73 ± 0.82	4.52 ± 0.65	<.001	.003	<.001	.064	.028	<.001
Q8: I believe that using the RTLS tag for locating patients within NCID is a good idea	3.63 ± 1.18	4.16 ± 0.76	3.85 ± 0.74	4.62 ± 0.62	.002	.551	<.001	.140	0.004	<0.001
Q9: I believe all healthcare workers in the NCID building should use the RTLS tag	3.44 ± 1.21	3.90 ± 0.89	3.79 ± 0.75	4.35 ± 0.80	.024	.245	<.001	.906	.012	.009
Q10: My peers think I should use the RTLS tag	3.00 ± 1.12	3.57 ± 0.83	3.29 ± 0.74	4.33 ± 0.91	.002	.389	<.001	.276	<.001	<.001
Q11: Patients in NCID (if aware of the functions of RTLS) think I should use the RTLS tag	3.04 ± 0.94	3.47 ± 0.86	3.19 ± 0.79	4.45 ± 0.79	.027	.843	<.001	.239	<.001	<.001
Q12: My seniors and supervisors think I should use the RTLS tag	3.44 ± 0.96	3.91 ± 0.85	3.63 ± 0.79	4.52 ± 0.68	.008	.677	<.001	.209	<.001	<.001
Q13: Overall, my organization supports the use of the RTLS tag	3.57 ± 0.91	4.05 ± 0.71	3.81 ± 0.64	4.50 ± 0.57	.001	.330	<.001	.229	.001	<.001
Factor 2: Perceived ease of use of RTLS tag (*α* = 0.8841)	−0.66 ± 1.15	−0.01 ± 0.88	−0.28 ± 0.79	0.76 ± 0.72	<.001	.157	<.001	.317	<.001	<.001
Q4: I find the RTLS tag easy to use for gaining access to the NCID building and wards	3.33 ± 1.12	3.75 ± 0.94	3.56 ± 0.92	4.17 ± 0.59	.040	.583	<.001	.623	.025	.004
Q5: I find the RTLS tag convenient to carry with me during work	2.29 ± 1.31	2.90 ± 1.10	2.85 ± 0.88	3.55 ± 1.05	.011	.063	<.001	.996	.001	.006
Q16: I find the RTLS tag compatible with my daily work routine (e.g. patient care)	3.13 ± 1.20	3.54 ± 0.96	3.33 ± 0.86	4.38 ± 0.76	.070	.729	<.001	.586	<.001	<.001
Q17: I find re-charging of the RTLS tag fast and simple	3.09 ± 1.13	3.66 ± 0.98	3.38 ± 1.10	4.52 ± 0.79	.007	.495	<.001	.350	<.001	<.001
Q18: I find re-charging of the RTLS tag convenient	2.89 ± 1.25	3.53 ± 1.02	3.10 ± 1.04	4.42 ± 0.87	.003	.751	<.001	.088	<.001	<.001
Factor 3: Privacy concerns on the use of RTLS tag (*α* = 0.8575)	−0.01 ± 1.11	−0.22 ± 0.88	−0.47 ± 0.75	0.79 ± 0.86	.586	.081	<.001	.417	<.001	<.001
Q19: I feel concerned about using the RTLS tag	3.22 ± 1.15	3.20 ± 0.89	3.00 ± 0.80	4.22 ± 0.89	.999	.653	<.001	.600	<.001	<.001
Q20: I find using the RTLS tag to monitor my hand hygiene compliance somewhat intimidating	3.35 ± 1.10	3.27 ± 1.01	2.90 ± 0.91	4.05 ± 0.96	.975	.126	.002	.131	<.001	<.001
Q22: I find the RTLS tag an invasion of privacy	3.30 ± 1.11	3.10 ± 1.00	2.85 ± 0.88	3.97 ± 0.88	.647	.114	.003	.453	<.001	<.001

##### Effort expectancy—physical inconvenience

One hundred and twenty-eight out of 198 feedbacks (64.6%) were about the physical inconvenience of the tags (20 physicians, 39 nurses, 13 AHPs, and 56 AAS), in terms of the dimension, durability, length of battery life, and the need to wear the tag consistently when in NCID.

###### Dimension

Thiry-four out of 75 nurses (45.3%) provided feedback on the bulkiness and weight of the tag, whilst other HCPs found it cumbersome to carry around.

“Tag is heavy, sometimes can be seen dangling down. And we have to pull it up [back to] position.” (Nurse 0101, 20 years in practice)

“Too cumbersome to bring around.” (Physician 0067, 9 years in practice)

###### Durability

In contrast, AAS provided the most feedback on the durability of the tag (38 out of 43 [88.4%]).

*“Easy to crack and damage.” (AAS 0273, 1.2 years in practice)*

“If have any cover to protect RTLS better, can prevent [it] from any damage.” (AAS 0252, 10.2 years in practice)

###### Length of battery life

The need for recharging was also an inconvenience for 3 physicians and 4 nurses.

*“Charging [is] inconvenient.” (Physician 0299, 2 years in practice)*

“Charging 2-monthly [usually forget to charge].” (Nurse 0105, 10 years in practice)

” It would be better if a charging port is made available in each ward so that it can be charged [up] easily.” (Nurse 0140, 3.7 years in practice)

###### Consistency of use

Two physicians and one AHP found the need for consistent use for the whole duration when providing clinical care to inpatients at NCID a challenge.

*“I think [the] main barrier for me is having to carry it or wear it all the time in clinical areas.” (Physician 0065, 15.5 years in practice)*

##### Performance expectancy—personal acceptance

In terms of personal acceptance of the RTLS tag, 6 out of 67 (9.0%) agreed that it was useful for contact tracing although 2 out of 67 (3.0%) expressed privacy concerns about being tracked. Some of the current technological limitations of the system have led to HCP’s doubts about the usefulness of the RTLS tag for hand hygiene reminders (38 out of 67 [56.7%]) and as access cards (20 out of 67 [29.9%]).

###### Perceived usefulness

Fifteen out of 27 nurses (55.6%) felt that the over-sensitivity of hand hygiene sensor was not useful for hand hygiene reminders, whilst AAS felt that the auditory cue was too soft to be effective (9 out of 11 [81.8%]).

*“[The] RTLS start[ed] to beep when [I walked] past the RTLS sensor, even when we had not contacted a patient. Even after we have done the first moment of hand hygiene before a procedure, and we pass by the sensor […] it starts to beep until the end of the procedure, which disturbs our work.” (Nurse 0229, 3.7 years in practice)*

*“Will still beep even when I practise[d] hand hygiene prior to speaking to [a] patient.” (AHP 0027, 2 years in practice)*

All categories of HCP (3 physicians, 7 nurses, 7 AHPs, and 3 AAS) felt that the poor detection of the RTLS tag by door access readers reduced its usefulness as an entry- and exit- access card.

*“Not ‘user-friendly’. When tapping RTLS, only the front side [of the tag] can [be detected] […] RTLS cannot be detected if there are staff pass[es] attach[ed] to it. Must be RTLS alone.” (AAS 0213, 5.5 years in practice)*

*“Agree with contact trace purpose but remove the incessant beeping please.” (Physician 0308, 6.5 years in practice)*

Whilst 3 out of 6 physicians (50.0%) appreciated the RTLS tag’s usefulness for contact tracing, one physician felt that the system was too expensive relative to its usefulness.

*“Huge waste of money for hand hygiene audit purposes. HCP are better trained than requiring a beeping device that cannot be intelligent enough to really accurately detect the compliance to hand hygiene.” (Physician 0308, 6.5 years in practice)*

###### Privacy concerns

A physician and a nurse expressed discomfort being tracked by the system and felt that it was an invasion of privacy.

*“Tracing staff location, feel uncomfortable.” (Nurse 0156, 1.7 years in practice)*

*“Invasion of privacy.” (Physician 0001, 28 years in practice)*

##### Technical support—enabling conditions

Two physicians and one nurse felt that better technical support could be provided for users.

*“Please provide us [with a] hotline [if] we need help” (Nurse 0091, 10 years in practice)*

## DISCUSSION

The majority (75.0%) of HCP working in inpatient wards managing COVID-19 patients at the national infectious disease referral center in Singapore were willing to carry the RTLS tag during routine care, with physicians (63.0%) being the least and AAS (90.0%) the most willing to use it. Regardless of the healthcare professional group, the HCP’s acceptance of the use of the RTLS tag in the hospital had the strongest association with the HCP’s willingness to use it. Acceptance to use a technology implies the behavioral intention of its use, which is influenced by perceived usefulness and social influence.^28^^,^^30^ By accepting the use of RTLS tag, the HCPs were motivated intrinsically due to self-perceived benefits of using the tag and encouraged by extrinsic factors such as organizational and peer influences in the use of the tag. Similarly, a Malaysian study showed that perceived usefulness and social influence had positive effects on the intention to adopt RFID technology by hospitals.^25^ Whilst the Malaysian study highlighted differences in factors influencing the adoption of RFID by the hospitals’ management and ground healthcare staff,^25^ our study did not find any difference in influencers of willingness to use the RTLS between categories of healthcare staff. Griffin et al^16^ also showed that low perceived usefulness of RTLS was associated with weaker intention to use the RTLS in the emergency department in the United States. One possible motivator for the acceptance of the RTLS tag among HCPs in NCID could be the perceived value of the tag for contact tracing, particularly in the context of the COVID-19 pandemic. This was supported by qualitative findings of physicians and AHPs expressing appreciation of the usefulness of the RTLS tag for contact tracing. The acceptance of the RTLS tag among these HCPs is expected to increase over time as the tags were shown to be accurate in identifying close contacts of COVID-19 patients during the pandemic.^12^

Whilst HCP perceived the tag to be useful for contact tracing, they were doubtful about the tag’s usefulness for hand hygiene reminders. Previous studies on the use of technology for hand hygiene reminders had faced similar challenges with user acceptance and satisfaction.^8^^,^^34^ However, there is value in providing user-specific auditory nudges to improve hand hygiene compliance. A previous mixed-methods study conducted by the team found that personal motivators and other enablers could result in a 60% increase in good hand hygiene compliance.^35^ Mask wearing and proper hand hygiene are the two main public health measures implemented during the COVID-19 pandemic, and therefore timely hand hygiene reminders are crucial. Tweaks in the system would be necessary to enhance the performance of the RTLS for its intended purpose to provide hand hygiene nudges and achieve its goal of enhancing hand hygiene compliance, including adjusting its detection sensitivities and the audibility of the auditory cue.

We further observed that the perceived ease of use of the RTLS tag was another independent predictor of the HCP’s willingness to use the tag. The observation was corroborated by findings in Malaysian hospitals where the perceived ease of use was found to be an important driver of healthcare and supporting staff’s intention to adopt RFID.^25^ Although AAS and nurses reportedly perceived that the RTLS tag was easy to use and were the most willing amongst HCP to use the tag in the hospital, they have provided qualitative feedback on the physical inconveniences including the weight, bulkiness, and durability of the tag. The mode of carrying the RTLS tag while carrying out their duties could have influenced how HCP perceived the tag’s ease of use. With the prevalent use of lanyards by AAS and clips by nurses, AAS were more likely to agree that the tag was convenient to be carried around during work while nurses found the tags to be heavy and bulky on their uniform, causing the collars of their uniforms to be dragged down by the tag. Hence, it is crucial to modify the RTLS tag’s portability on uniforms and in pockets to increase convenience for HCP who do not carry the tag with a lanyard.

To make the tag more wearable and portable, many HCP have suggested making the tag lighter and thinner, and to incorporate staff identity cards into the same card. Previous studies have shown that multi-functional technologies with default systems to facilitate intended behaviors of users could increase compliance with the use.^36^ However, the more functions the RTLS tag were to incorporate, the more challenging it would be to reduce its size and weight. The design and creation of better accessories could make the tag more wearable to increase compliance and sustained use of the tag. As nurses spend the most time in the inpatient wards, improving the accessories to enable the tag to be well clipped to the nurses’ uniforms and not be dragged down from prolonged use is crucial (Table 3). Co-option of end-users of the technology (with representatives from each of the HCP groups) in the design of the accessories, is key to the successful and sustained use of the RTLS tag.^37–39^

Furthermore, the various functions of the tag would need to be explicitly communicated to staff to increase acceptance and compliance. AHPs (70.2%) were least aware that their RTLS tags were tagged to their individual identities, whilst nurses (80.8%) were least aware that the tags served as contact tracing tools and physicians (75.6%) that the RTLS tag could prompt and remind them of the need for hand hygiene. Frequent communications to HCP on the functions of the RTLS tags and promotion of the social norm of full compliance with carrying the tags during patient care in the hospital for their and their patients’ safety during the COVID-19 pandemic is crucial.

Due to the current-day limitations in technology, the tag would require to be manually charged every 2–3 months. Provision of chargers in the inpatient wards and during department meetings would increase the convenience for charging and serve as reminders for charging. Furthermore, email reminders could also be triggered and automatically sent to HCP to remind them to charge the tags, whenever batteries run low. As with the development of any new habit, it is anticipated that it can take up to 8 months (254 days) before the RTLS tag becomes a way of life for HCP in the hospital.^40^

Interestingly, privacy concerns did not emerge as a significant factor associated with HCP willingness to use the RTLS tag, although HCP had shared concerns about the invasion of privacy and discomfort with one’s location being tracked.

*“Tracing staff location, feel uncomfortable.” (Nurse 0156, 1.7 years in practice)*

“Invasion of privacy.” (Physician 0001, 28 years in practice)

Furthermore, while AAS were more likely to express concerns on privacy, they were the most willing among the HCP to use the RTLS tag. The importance of uniform compliance could have motivated AAS to use the RTLS tag in spite of their privacy concerns. Nonetheless, it is critical to inform staff of the data protection policies and processes pertaining to the data captured by the RTLS and to reassure staff that the use of the data was solely for the purposes of ensuring patient safety and for contact tracing to prevent nosocomial transmission of an infectious disease. The reassurance of data security and HCP’s trust in the system is crucial especially when there is a need for full compliance by staff in the use of the RTLS tag during the COVID-19 pandemic. Contact tracing technologies have been identified as one of the key strategies for life to return to some form of normalcy in the next phase of the COVID-19 pandemic.^15^ More urgent than ever is the need to optimize the utilization of technologies such as RFID.

### Strengths and limitations

The study had several strengths. First, it had a high participation rate of >70%, with a good representation of physicians, nurses, AHPs, and AAS who provide inpatient care at the hospital and use the RTLS tags routinely. Second, the study is anonymous and completed surveys were deposited at the convenience of HCP into sealed boxes. As such, the responses in the questionnaire were highly likely to be authentic. Furthermore, qualitative methods were embedded to provide deeper insights into the experience and concerns of HCP with the use of the RTLS tags. The strengthening of findings using mixed methods has provided robust findings on which to base interventions to enhance user experience and compliance with the technology.

However, the study could be limited by the inability to assess for factors not collected by the survey that could influence the willingness of HCP to use the RTLS tag. Nonetheless, the key constructs that have been internationally established to be associated with technology adoption, including specific ones for the use of RFID in healthcare and other settings, have been included in the study questionnaire. Potential confounding due to age, gender, and context experience have also been addressed in the multivariable logistic regression analysis.

## CONCLUSION

Regardless of the healthcare professional group, the HCP’s acceptance of the use of the RTLS tag in the hospital had the strongest association with the HCP’s willingness to use it. Furthermore, HCP’s perceived ease of use of the tag also positively influenced their willingness to use it. More can be done to improve communications on the intentions of the technology and to enhance the convenience for HCP’s sustained use of the RTLS tag, to optimize its usefulness during the COVID-19 pandemic.

## AUTHORS’ CONTRIBUTIONS

H.G. analyzed and interpreted the quantitative data and drafted the manuscript. Z.H. assisted with interpreting study data and drafting the manuscript. J.Y.P.Y. analyzed and interpreted the qualitative data, and provided inputs for the manuscript. Y.W. prepared the questionnaires, collected the responses, assisted with analyzing and interpreting the qualitative data, and provided inputs for the manuscript. A.C. conceived the study, provided overall direction and planning for the study, analyzed and interpreted the data, and critically revised the manuscript.
